# Patients’ knowledge about dental emergencies, COVID-19 transmission, and required preparations in dental settings

**DOI:** 10.1371/journal.pone.0301460

**Published:** 2024-04-18

**Authors:** Mohammad Reza Khami, Mahsa Karimi, Morenike Oluwatoyin Folayan, Ahmad Reza Shamshiri, Heikki Murtomaa

**Affiliations:** 1 Research Center for Caries Prevention, Dentistry Research Institute, Tehran University of Medical Sciences, Tehran, Iran; 2 Community Oral Health Department, School of Dentistry, Tehran University of Medical Sciences, Tehran, Iran; 3 Dental Students’ Scientific Research Centre, School of Dentistry, Tehran University of Medical Sciences, Tehran, Iran; 4 Department of Child Dental Health, Obafemi Awolowo University, Ile-Ife, Osun State, Nigeria; 5 Oral Public Health Department, University of Helsinki, Helsinki, Finland; Shahid Beheshti University of Medical Sciences School of Dentistry, ISLAMIC REPUBLIC OF IRAN

## Abstract

**Introduction:**

The Coronavirus disease 2019 (COVID-19) pandemics negatively affected the delivery of dental care. The study objective was to assess the knowledge of emergency dental treatments, the transmission routes of COVID-19 in the dental settings, necessary precautions to prevent disease transmission, and the associations between demographic factors and the mentioned domains among Iranian dental patients.

**Methods:**

This was a cross-sectional study conducted in October 2021. A systematic random sampling approach was used to select 244 participants who had sought services at the dental clinic of Tehran University of Medical Sciences before and during the pandemic. Data was collected using a combination of interviewer-administered and self-administered questionnaire. Three backward stepwise multiple logistic regression analyses were conducted to determine the associations between background factors (age, sex, education level, living status, history of dental visits, history of COVID-19 vaccination, and past COVID-19 infection) and knowledge about emergency dental treatments, knowledge about COVID-19 transmission routes, and knowledge about necessary preparations in dental settings.

**Results:**

The mean (SD) scores for knowledge of emergency dental treatments, COVID-19 transmission routes, and essential preparations in dental settings measured on a 100-point scale were 77 (15.4), 84.2 (12.3), and 93.3 (12.1), respectively. Good knowledge of emergency dental treatments was associated with being under 55 years old (p = 0.03). Good knowledge of COVID-19 transmission routes was associated with living with individuals at a high risk of COVID-19 (p = 0.014) and having received the COVID-19 vaccine (p = 0.013). After adjusting for age, among participants aged 30 years and older, good knowledge of necessary preparations in dental settings was associated with being female (p = 0.012) and having received the COVID-19 vaccine (p = 0.001).

**Conclusions:**

Patients who sought care at the dental clinic of Tehran University of Medical Sciences had good knowledge about the transmission routes of COVID-19 and the required preparations in dental settings, and limited knowledge about dental emergency treatments.

## Introduction

The delivery of dental care was significantly disrupted by the Coronavirus Disease 2019 (COVID-19) pandemic [1]. This measure aimed to mitigate the risk of Severe acute respiratory syndrome coronavirus 2 (SARS-CoV-2), the virus responsible for COVID-19, which can be transmitted through respiratory droplets, proximity, or contact with contaminated surfaces [2]. Dental procedures, generating aerosols, pose a potential risk of infection transmission among patients and dental staff. Therefore, it was imperative to implement precautionary measures in dental settings to curb the spread of diseases [3].

To address this concern, the World Health Organization (WHO), Centers for Disease Control and Prevention (CDC), American Dental Association (ADA), and other regulatory bodies issued clinical guidelines focused on controlling the transmission of COVID-19 in dental settings. In the early stages of the pandemic, these guidelines recommended restricting dental treatments to emergency cases [4].

Restricting dental services to emergency procedures during and after the epidemic has the potential to compromise public oral health [5]. Patients face the critical task of distinguishing between urgent and non-urgent dental care needs and must make informed decisions about seeking treatment [6]. While forgoing a dentist visit during a dental emergency can jeopardize oral health, unnecessary visits for non-emergency conditions may increase the risk of COVID-19 infection and negatively impact overall health [7].

There is scarce information regarding dental patients’ knowledge of emergency dental treatments. Despite studies being conducted on the general public’s awareness of COVID-19 transmission [8, 9], there is, to the best of our knowledge, a lack of research assessing dental patients’ knowledge about transmission routes and necessary preparations in dental settings to reduce the risk of COVID-19 transmission. Therefore, our study aimed to evaluate the understanding of Iranian dental patients concerning emergency dental treatments, transmission routes of COVID-19 in dental settings, and the necessary preparations to prevent disease spread in dental settings. Furthermore, we assessed the associations between background factors (age, sex, education level, living status, history of dental visits in the last year, COVID-19 vaccination status, and past COVID-19 infection) and their knowledge in these domains.

## Materials and methods

This was a cross-sectional study conducted between October 1st and October 30th, 2021. The participants were patients who had sought services at TUMS dental clinic. Patients below 18 years old and those unable to comprehend the Persian language were excluded from the study.

A preliminary investigation was conducted to determine the standard deviation (SD) for the COVID-19 preparation knowledge score. The minimum sample size for the study was calculated to be 236 participants based on a SD for the COVID-19 preparation knowledge score of 31.41 measured on a 100-point scale, a significance level (α) of 0.05 and a margin of error of 4 points [10].

Participants were chosen through a systematic random sampling method. Initially, a list of phone numbers belonging to patients who had visited the clinic during 2019 (representing the pre-COVID-period), 2020 and 2021 (representing the post-COVID-period) was compiled. To ensure an equal representation of participants from the pre- and post-COVID-19 periods, patients from 2019 constituted 50% of the sample size. The remaining 50% was evenly distributed between patients from 2020 and 2021, with both years representing 25% of the total sample size.

To enlist participants, researcher (MK) reached out to individuals who met the eligibility criteria through individual phone calls. Participants were introduced to the study and had the option of receiving a link to self-administer the questionnaire or for an interviewer to administer the questionnaire. For interviewer-administered questionnaires, the researcher presented the consent page, and upon receiving verbal consent, marked the corresponding box on the form. The telephone interview ensued, with the interviewer directly inputting the participant’s responses into the Google Form. For self-administered questionnaire, the link to the online questionnaire was shared with the participant through platforms they preferred such as Telegram, WhatsApp, or emails. The forms could only be submitted after all questions had been answered.

Data were collected using validated questionnaire [11] accessible on Google Forms (https://forms.gle/JVTo7tucZRx9Nsu38) in Persian. Form settings were configured to maintain response confidentiality, ensure respondent anonymity, and allowed each participant to submit only one form. The survey’s consent page outlined the study’s purpose, stressed voluntary participation, assured participants of response confidentiality, and sought their consent to partake in the study through a tick on a checkbox. Those who did not consent to study participation were thanked and exited from filling the form.

The questionnaire utilized in this study underwent a thorough and rigorous process of development and validation. The researchers formulated the various domains and compiled the items after conducting an extensive review of existing literature. A group of experts in community dentistry assessed each item of the questionnaire for its essentiality, relevance, clarity, and simplicity. These assessments were then utilized to evaluate the validity of the tool using the Item Content Validity Index (I-CVI) and the Scale Content Validity Index (S-CVI/Ave). Furthermore, a pilot study was conducted to ascertain the reliability of the questionnaire by calculating the Cronbach’s alpha. To enhance the face validity of the questionnaire, feedback from both experts and laypeople was taken into consideration. The S-CVI/Ave of the tool ranged from 0.63 to 0.99 and the Cronbach’s alpha ranged from 41.1 to 87.6.

The questionnaire employed in this study (S1 File) gathered data on the sex assigned at birth, age at the last birthday, educational attainment (primary school, secondary school, high school diploma, associated degree, bachelor’s degree, masters or higher degrees), living arrangements (alone, with no high-risk or elderly individuals, with high-risk or elderly individuals), COVID-19 vaccination status (one shot, two shots, none), COVID-19 status (no positive test, at least one positive test), and dental visit history in the last 12 months (Yes, non-emergent problem; Yes, urgent problem; No, due to COVID-19 despite oral issues; No, no problem; Yes, routine check-ups). For logistic regression analysis, the information on COVID-19 vaccination status was dichotomized into "yes" (one shot, two shots) and "no" (no shot) and the information on living status was dichotomized into "low risk" (alone, with no high-risk or elderly individuals) and "high risk" (with high-risk or elderly individuals). This demographic information was the independent variable for the study. It should be noted that a high-risk individual is one who, due to underlying medical conditions, is more susceptible to comorbidities arising from contracting COVID-19 [12].

The study had three dependent variables: patients’ knowledge of dental emergencies (nine questions), knowledge of COVID-19 transmission routes in dental settings (eight questions), and knowledge of required preparations in dental settings (11 questions). The score for each domain was calculated as the percentage of correct answers to the items within that domain for each respondent. The percentage of the correct answers to the items of each domain served as the domain’s score for each respondent.

The nine questions assessing knowledge of dental emergencies comprised a list of common dental procedures [13]. Participants were tasked with identifying which of the listed treatments qualified as emergency procedures by responding true or false to each listed item. The questions on COVID-19 transmission routes and necessary preparations in dental settings were prepared following a systematic search of the literature including public health guidelines. Respondents also had to check a true or false response to each of the questions. Correct responses were given a score of 1 while incorrect responses were given a score of 0. Each respondent’s score was converted to a 100%.

Regarding the knowledge of COVID-19 transmission routes and required preparations in dental settings, the researchers decided to categorize participants who made zero or one mistake as having good knowledge; since these sections were closely related to general knowledge of COVID-19, which was widespread during the pandemic. However, knowledge of emergency dental treatments had been exclusively related to the dental profession before the COVID-19 pandemic and emerged as a novel required knowledge for the general public during this time. As a result, the researchers decided to categorize participants who made up to two mistakes in this section as having good knowledge.

For data analysis, responses collected through Google Forms were downloaded into a Microsoft Excel 2013 file. Subsequently, the data underwent thorough cleaning and coding processes before being imported into IBM SPSS Statistics version 21 for Windows (IBM Corp, Armonk, New York, 2012). Means and standard deviations of the knowledge domains were reported as the primary outcome. Three backward stepwise multiple logistic regression analyses were conducted to assess for the associations between the dependent and the independent variables as the secondary outcomes. Odds ratios (OR) and 95% confidence intervals (95% CI) were calculated, with a significance level set at 5%.

Ethical approval for the study was obtained from the Research Ethics Committee at Tehran University of Medical Sciences (TUMS) (IR.TUMS.DENTISTRY.REC.1400.117).

## Results

Out of the 378 eligible participants, 244 participated in the survey, resulting in a response rate of 64.5%. Among the participants, 223 (91.4%) opted for an interviewer-administered questionnaire through a phone interview. Table 1 presents a demographic overview of the participants. There were 137 (56.1%) female respondents, 32 (13.1%) had education levels up to primary school, 111 (45.5%) had visited the dental clinic for non-emergency care in the last 12 months, and 86 (35.2%) had received a single shot of the COVID-19 vaccine.

**Table 1:** Characteristics of a sample of Iranian dental patients (N = 244).

Age (years), M* ± SD (range)	42.9 ± 14.2 (18–88)
**Sex, N (%)**	
Female	137 (56.1)
Male	107 (43.9)
**Education level, N (%)**	
Up to Primary	32 (13.1)
Secondary	18 (7.4)
High school diploma	71 (29.1)
Associated	14 (5.7)
Bachelor	72 (29.5)
Masters or higher	37 (15.2)
**Living status, N (%)**	
Live alone	11 (4.5)
Live with neither high-risk for COVID-19 nor old people	137 (56.1)
Live with a high-risk for COVID-19 or old people	96 (39.4)
**Dental visits, N (%)**	
Yes, because of a non-emergent problem.	111 (45.5)
Yes, because of an urgent problem.	56 (23)
No, because of COVID-19, although I had some problems in my mouth.	32 (13.1)
No, because I did not have any problem.	36 (14.8)
Yes, routine check-ups.	9 (3.7)
**COVID-19 vaccine, N (%)**	
One shot	86 (35.2)
Two shots	79 (32.4)
No shot	79 (32.4)
**COVID-19 status, N (%)**	
No positive test	197 (80.7)
At least one positive test	47 (19.3)

*: Mean

Table 2 shows that the mean (SD) knowledge scores for emergency dental treatments, COVID-19 transmission in dental settings, and required preparations in dental settings was 77.0 (15.43), 84.2 (12.35), and 93.3 (12.11), respectively.

**Table 2:** COVID-19 knowledge of dental emergencies, the transmission of COVID-19 in dental settings, and required preparations in dental settings among a sample of Iranian dental patients by background variables (N = 244).

Variables Category*	Knowledge of emergency (M±SD)	Knowledge of transmission (M±SD)	Knowledge of required preparation (M±SD)
Gender			
Male	77.5±14.2	85.0±11.8	92.6±12.9
Female	76.6±16.4	83.6±12.8	93.8±11.5
Education			
Up to Primary	75.0±12.0	82.0±12.3	93.7±8.5
Secondary	71.0±18.3	82.6±14.9	97.0±6.2
High school diploma	78.6±15.2	82.9±12.7	93.8±15.5
Associated degree	73.0±18.8	82.1±10.6	96.7±4.5
Bachelor’s degree	76.8±15.0	85.9±11.9	92.3±11.6
Master’s degree or higher	80.2±16.2	86.8±11.8	90.7±12.2
Living Status			
Alone	80.8±18.6	84.1±12.6	95.0±7.5
Not with high-risk	76.4±16.3	83.1±13.4	92.4±14.1
With high-risk	77.3±13.8	85.8±10.5	94.3±9.2
Dental Visit			
No, didn’t have any problem	74.7±15.4	84.4±12.5	95.7±5.9
No, because of COVID-19	79.5±12.3	86.7±8.9	93.2±8.6
Yes, an urgent problem	75.0±16.4	83.9±13.0	95.1±9.2
Yes, a non-emergent problem or check-up	77.9±15.7	83.6±12.8	91.7±15
COVID-19 Vaccine Shots			
Yes	77.6±15.3	85.7±11.4	94.2±9.8
No	75.7±15.7	81.2±13.7	91.4±15.7
COVID-19 Test Results			
At least one positive test	75.4±15.2	84.6±9.5	92.1±14.1
No positive test	77.3±15.5	84.1±13	93.6±11.6
**Total Participants**	**77.0±15.4**	**84.2±12.3**	**93.3±12.1**

*: Categories with less than 20 participants were combined with one of their neighbor categories having a more similar score in that domain.

Table 3 displays the associated variables with each knowledge domain. Since the data for all knowledge scores was not normally distributed, we utilized categorical knowledge variables, including “good” and “poor” knowledge. Poor emergency knowledge was associated with being 55 years old or older (p = 0.030). Poor transmission knowledge was associated with living alone or with COVID-19 low-risk individuals (p = 0.014) and not being vaccinated (p = 0.013). As age interacted with other associated variables in the preparation knowledge regression model, it was considered an effect modifier. This means that younger and older participants were separately analyzed regarding the associated variables within this knowledge domain. Regarding 30-year-old or younger individuals, poor preparation knowledge was associated with being male (p = 0.012) and not being vaccinated (p = 0.001).

**Table 3:** Background characteristics associated with dental patients’ (N = 244) knowledge.

Domain	Variable*	Odds ratio	95% Confidence Interval	p-value
Emergency	Age (years)			
	Under 55	Ref.	-	-
	55 or older	0.512	0.280 to 0.937	0.030
Transmission	Living status			
	Alone or with COVID-19 low-risk individuals	0.487	0.274 to 0.864	0.014
	With COVID-19 high-risk individuals	Ref.	-	-
	Vaccine status			
	Vaccinated	Ref.	-	-
	Not vaccinated	0.488	0.277 to 0.857	0.013
Preparation	**Age 30 or older (N = 192)** ^†^			
	Gender			
	Male	0.291	0.111 to 0.761	0.012
	Female	Ref.	-	-
	Vaccine status			
	Vaccinated	Ref.	-	-
	Not vaccinated	0.192	0.075 to 0.493	0.001
	**Age under 30 (N = 52)** ^†^			
	-			

Multiple logistic regression by use of the backward stepwise method

*: Only significantly associated variables (p < 0.05) that remained in final regression models of each domain are presented in the table

^†^: Effect modifier; Ref: Reference category which other categories of a particular variable were compared within the regression model.

## Discussion

Our study results suggest patients who attend the dental clinic at TUMS have good knowledge of emergency dental treatments, COVID-19 transmission, and required preparations in dental settings. However, notable distinctions were observed. Older Iranian patients exhibited comparatively poorer knowledge of emergency dental treatments when compared to younger Iranian patients; and respondents living alone or with individuals at low COVID-19 risk, as well as those unvaccinated against COVID-19, demonstrated poorer knowledge about COVID-19 transmission routes in dental settings. Furthermore, among individuals aged 30 and above, males and those who had not received COVID-19 vaccination had poorer knowledge about required preparations in dental settings to prevent COVID-19 transmission.

One of the strengths of this study is its contribution to the body of knowledge regarding public awareness of the attributes of dental emergencies, an area with limited coverage in English literature. While the significance of this issue may not be immediately evident in day-to-day life, it assumes critical importance in specific scenarios, such as infectious disease outbreaks. In such situations, a proper understanding of dental problems becomes essential, as both unnecessary referrals and non-referrals during emergencies can potentially lead to complications. In addition, the high internal consistency, use of reliable scales, the use of regression analysis to control for variable effects, high statistical power attributable to an appropriate sample size, and the utilization of effect modification were other strengths of the study.

Prior to the onset of the COVID-19 pandemic, awareness of emergency dental treatments was primarily confined to the dental profession and subsequently emerged as a newfound essential knowledge for the public during this period. In addition, recognizing the dynamics of COVID-19 transmission and the essential precautions in dental settings can empower patients to evaluate dental facilities for safety and this awareness aids in choosing facilities prioritizing patient safety. Refusing to attend dental clinics which patients recognize their lack of sufficient precautions against COVID-19, is a good case in point. Public expectations can also foster the implementation of heightened safety protocols in dental care [14].

In the current study, younger participants had better knowledge of emergency dental treatments compared to their older counterparts. This finding corroborates the results of the study by Von dem Knesebeck et al. [15], where younger individuals in the German population found it easier to evaluate a health problem as a medical emergency. The more frequent use of social media by younger population could explain their better understanding of urgent medical or dental problems. We strongly advocate for patients to be well-informed about dental emergencies, and ideally, the findings in this section suggests correcting the elderlies’ point of view due to the inaccuracies.

The influx of information on social media heightened public awareness regarding the transmission routes of COVID-19 [16]. The findings of current study indicate that a more comprehensive understanding of COVID-19 transmission routes is linked to vaccination status. Previous investigations into infectious disease epidemics, including the COVID-19 pandemic, had demonstrated that knowledge and awareness of a disease can serve as a motivating factor for the public to adopt preventive behaviors [17–19], such as opting for vaccination. Moreover, the higher levels of knowledge observed in vaccinated participants in this study may be attributed to the prioritization of vaccination for high-risk individuals and healthcare workers during the data collection period in Iran [20]. Additionally, living arrangements are associated with transmission knowledge. Given the prevalent concern among individuals about transmitting COVID-19 to their loved ones rather than contracting the disease themselves [21, 22], those residing with high-risk individuals are more likely to actively seek information and possess greater knowledge about transmission routes.

During the COVID-19 pandemic, the World Health Organization (WHO) and the Centers for Disease Control and Prevention (CDC) issued stringent guidelines on cross-infection control in dentistry [23, 24], urging dentists to implement these protocols for the protection of both patients and staff. Among participants aged 30 and above in the present study, females demonstrated a higher level of knowledge about required preparations in dental settings, consistent with their general propensity for greater knowledge in prevention-related subjects [25–28] and a heightened interest in health matters compared to males [29, 30]. Furthermore, a positive association between vaccination status and higher knowledge of required preparations in dental settings was observed, mirroring the findings from the analysis of knowledge of transmission of COVID-19 in the dental settings.

Although the present study indicates that dental patients possess satisfactory knowledge regarding COVID-19 transmission and prevention, it is essential to maintain efforts in educating both patients and dental staff about preventive measures. Specific attention should be given to aspects unique to dental settings, such as emphasizing the significance of pre-appointment screening and the use of mouthwash before dental procedures. Furthermore, there is a necessity to consistently augment and extend this knowledge to less informed groups of patients by actively promoting continuous education and awareness in the dental clinics thereby ensuring a secure environment for both patients and staff. The study has a few limitations. First is the lower than optimal response rate of 80% which may limit the generalizability of the study finding [31]. Additionally, the sampling framework is limited to patients attending dental clinic at TUMS. These patients represent individuals with a higher socioeconomic status, as those seeking dental services in Iran typically have better socioeconomic status than those who do not [32]. This discrepancy may account for the high level of knowledge observed in this study, and thus, caution should be exercised when extrapolating these findings to the broader population. Furthermore, self-reported vaccination and COVID-19 history introduce socially desirable responses from participants, necessitating careful consideration during result interpretation. Additionally, the use of two data collection methods, telephone interviews and online questionnaires, may result in less comparable outcomes between participants who completed the survey through different methods. However, this issue is not significant due to the minimal number of respondents who participated through self-administered questionnaires. Despite the fact that data gathering was performed during the peak of the pandemic and this could limit the generalizability of the findings to today’s situation, some findings from the study can offer insights for planning in the context of future pandemics and crises, such as COVID-19, within similar populations.

## Conclusions

Patients who attended the dental clinic at TUMS had acceptable knowledge about the transmission routes of COVID-19 and the required preparations, and limited knowledge about the characteristics of dental emergency treatments. Age, sex, COVID-19 vaccination, and living status seem to be associated with knowledge of participants. This situation emphasizes the importance of paying attention to the dissemination of scientifically justified information and public education for future crises. However, the plausibility of these evidences needs to be studied further.

## Supporting information

S1 File Study questionnaire. (DOCX)

S1 Data set Study data set. (SAV)

## List of abbreviations

COVID-19: Corona Virus Disease 2019
SARS-CoV-2: Severe acute respiratory syndrome coronavirus 2
WHO: World Health Organization
CDC: Center for Disease Control and Prevention
ADA: American Dental Association
SD: Standard Deviation
MOE: Margin of Error
OR: Odds Ratio
CI: Confidence Interval
M: Mean
