# Supplementary material for: Patients’ knowledge about dental emergencies, COVID-19 transmission, and required preparations in dental settings
Source: PLoS One. 2024 Apr 18;19(4):e0301460. doi: 10.1371/journal.pone.0301460 (PMC11025918; doi:10.1371/journal.pone.0301460)
Supplement: S1 File — (DOCX) [file pone.0301460.s001.docx]

**
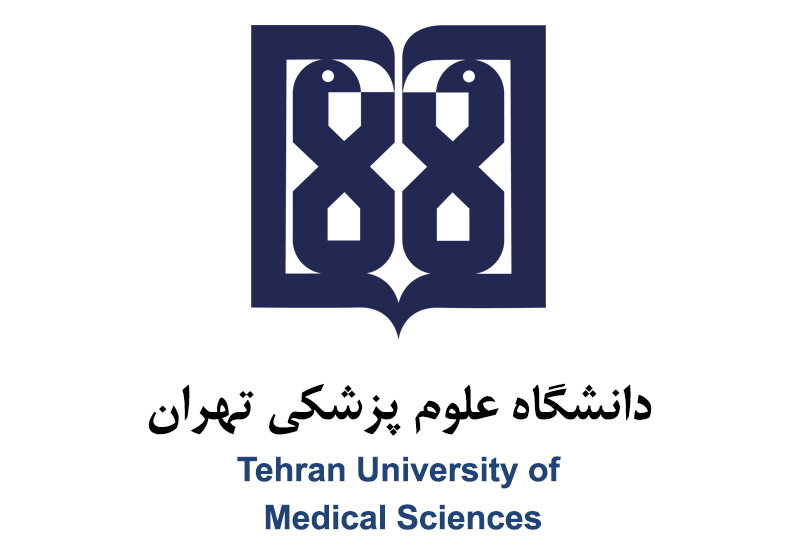
Tehran University of Medical Sciences, School of dentistry**

Knowledge and attitude of dental patients regarding COVID-19 infection in dental settings

**1. Demographic information**

1. Please select the method by which you are completing the questionnaire.

Online link□ Phone interview□

2. Gender: Male □ Female □

3. Year of birth: …………….

4. Highest academic degree:

No formal education □ Reading and writing □

Primary education □ Secondary education □ High school diploma □

Associated degree □ Bachelor □ Master □ Doctorate□ Post doctorate □

5. Do you share a residence with someone to whom you are concerned about transmitting Corona virus?

I live alone. □

I live with people who are neither at high risk nor are over 65 years old.□

I live with people who are at high risk (have an underlying disease and/or over 65 years of old).□

6. Have you visited a dentist in the last year?

Yes, because of a routine check-up. □

Yes, because of a none urgent problem. □

Yes, because of an urgent problem. □

No, because of Corona virus, although I had some problems in my mouth.□

No, because I did not have any problem. □

Never visited a dentist. □

7. Have you been vaccinated against the Corona virus?

Yes, one shot. □ Yes, two shots. □ No. □

8. Have you tested positive for Corona virus?

Yes, I have tested positive for Corona virus once or more.□

No, I have never tested or my test result was negative.□

**2. Awareness of emergency dental treatments:**

Which of the following conditions would you consider to be emergencies that require dental visit during the Corona virus pandemic? (You can choose more than one statement.)

1. Painful swelling in or around your mouth and cheek □

2. Regular visits for braces and fixed orthodontic appliances (devices used in dentistry that align and straighten teeth and help position them with regard to a person's bite.)□

3. Treatment of cavities that aren’t painful □

4. Regular visits for exams, cleanings, and x-rays □

5. Denture adjustment for people under radiotherapy and/or chemotherapy □

6. Broken or knocked out tooth □

7. Gum infection with pain or swelling □

8. Pain in a tooth, teeth or jaw bone □

9. Any lesion (painful or not) in the mouth persisting for two weeks or more □

10. None of the above □

**3. Knowledge on Corona virus transmission routes in dentistry**

Which of the following do you think can potentially transmit Corona virus infection in dental settings? (Please select all that apply)

1. Inhaling respiratory droplets produced when an infected person coughs, sneezes, or talks□

2. Through intact skin (healthy skin in which there are no breaks, scrapes, bruises etc.) □

3. Contact with contaminated dental instruments and/or environmental surfaces □

4. Talking without a mask □

5. Close contact with other asymptomatic patients □

6. Inhaling fine droplets formed during dental procedures □

7. Contacts of oral, nasal, and eye mucous membrane with surfaces contaminated with oral fluids of other patients □

8. Being in close contact (less than 2 meters or 6 feet distance) with an infected person (patients, dentist, staff) for more than 15 minutes in a closed space without mask □

9. None of the above □

**4. Awareness of required preparations in dental practice settings**

Which of the following do you think required preparations in dental practice settings? (Please select all that apply)

1. Adequate air conditioning (air flow or air circulation) in both waiting rooms and treatment rooms □

2. Screen staff and patients for flu-like symptoms, respiratory distress or fever as daily routine before they enter to the office/clinic □

3. Ensuring a spatial sitting distance of at least 6 feet (2 meters) among patients in the waiting area □

4. Enforce isolation of patients infected with/suspected of Corona virus infection in the waiting area □

5. Limit (or restrict) dental treatments for those infected with/suspected of Corona virus to only emergency procedures □

6. Use effective pre-procedural antimicrobial mouthwash □

7. Clean and disinfect public areas frequently, including door handles, chairs, and bathrooms□

8. Post visual alerts/ signs of instructions for hand and respiratory hygiene, and cough etiquette □

9. Use of full personal protective equipment including masks, gloves, gowns, and goggles or face shields by dentists and nurses □

10. Instruct patients to call before their appointment if they have respiratory symptoms so that staff can be prepared to care for them when they arrive □

11. Complete tele/online evaluation before the first visit.□

12. None of the above □
